# A Case Report of Massive Intraperitoneal Hemorrhage from Rare Cornual Pregnancy

**DOI:** 10.5811/cpcem.2021.10.54388

**Published:** 2022-01-28

**Authors:** Brandon M. Carius, Edward J. Houston, Stephen P. Griffith

**Affiliations:** Brian D. Allgood Army Community Hospital, Department of Emergency Medicine, Camp Humphreys, Republic of Korea

**Keywords:** ectopic, abdominal pain, pregnancy, case report

## Abstract

**Introduction:**

A cornual pregnancy describes a rare ectopic location positioned within the myometrium next to the fallopian tube, which can be difficult to find on traditional ultrasound imaging. Given its location and the stretch within the uterine wall, cornual pregnancies can progress for weeks prior to diagnosis. Ruptures can, therefore, be catastrophic with disproportionally high maternal mortality rates compared to other ectopic pregnancies.

**Case Report:**

A 34-year-old female recently treated with methotrexate for ectopic pregnancy presented to the emergency department (ED) for acute onset of lower abdominal cramping without vaginal bleeding. She arrived clinically stable and quickly decompensated with witnessed syncope in the ED, prompting point-of-care ultrasound showing free fluid in the abdomen. The patient was taken for emergent surgery by obstetrics while receiving transfusion of blood products for suspected ruptured ectopic pregnancy. A fetus estimated to be 10 weeks of age was discovered in the left cornual region. Approximately two liters of intraperitoneal blood were drained without complication.

**Conclusion:**

Cornual pregnancy is a difficult to diagnose but potentially disastrous type of ectopic pregnancy due to massive hemorrhage. Emergency clinicians should be aware of this condition given its rare occurrence but potentially catastrophic outcomes.

## INTRODUCTION

Pregnancy complications are frequently seen in the emergency department (ED) setting, where evaluation includes hemodynamic stability and pregnancy location and viability. While modern ultrasound (US) can help locate pregnancy and assess viability, some may be difficult to locate. Cornual pregnancy is a rare type of ectopic pregnancy rarely described in emergency medicine literature. Because it may go unidentified for weeks cornual pregnancy demonstrates high maternal mortality because of its delayed diagnosis and massive hemorrhage with rupture. We report a case of this rare type of ruptured ectopic pregnancy presenting as stable abdominal pain with sudden hemodynamic instability due to severe intraperitoneal hemorrhage.

## CASE REPORT

**Image 1:**
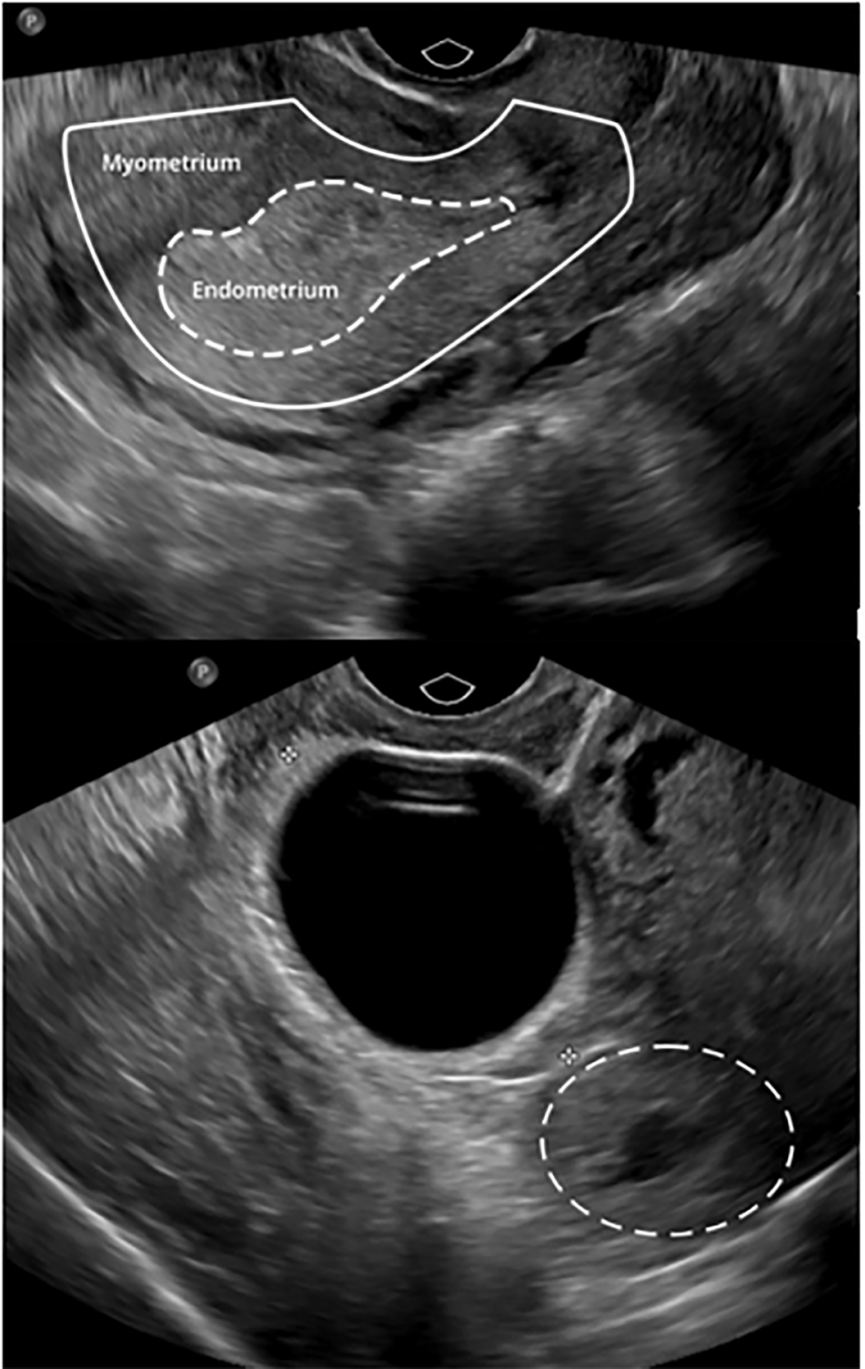
Initial transvaginal ultrasound six weeks prior to presentation, revealing an empty uterus (top) and a “cystic area” measuring 2.3 × 2.5 × 3.2 centimeters in the left cornual region (bottom, dashed circle).

A 34-year-old gravida four, para two female with history of two ectopic pregnancies arrived at the ED complaining of 20 minutes of left-sided lower abdominal pain with four episodes of non-bloody emesis. She denied other symptoms, including vaginal bleeding. Initial medical history included two previous ectopic pregnancies; the first, approximately six months prior to arrival at the ED, resolved with intramuscular methotrexate. Her second ectopic pregnancy was suspicious for cornual pregnancy based on history and US at seven weeks estimated gestational age (Image 1) but had been officially diagnosed by obstetrics as an ectopic pregnancy of unknown location and deemed resolved after intramuscular methotrexate one month prior to arrival. After initial quantitative beta-human chorionic gonadotropin (βhCG) of over 16,000 milli-international units per milliliter (mIU/mL) (normal hCG range: <3 mIU/mL), her levels fell to 1700 mIU/mL two weeks prior to arrival.

Triage vital signs were significant for blood pressure (BP) of 86/51 millimeters mercury (mm Hg) but were otherwise within normal limits, including a heart rate (HR) of 85 beats per minutes (bpm). Physical examination revealed voluntary guarding of the abdomen and only moderate tenderness of the left lower quadrant. A one-liter saline intravenous bolus provided transient BP improvement to 108/58 mm Hg with other vital signs within normal limits. While preparing for an abdominal and pelvic computed tomography, initial complete blood count (CBC) found stable hemoglobin and hematocrit of 12.0 grams/deciliter (g/dL) (reference range: 12.0 – 16.5 g/dL) and 36.1% (reference range 36.0 – 49.5%), respectively. Approximately five minutes after last vital signs were taken, the patient stood to provide a urine sample and nursing witnessed sudden diffuse pallor immediately followed by syncope lasting less than 60 seconds. Repeat vital signs revealed BP of 68/38 mm Hg and HR of 108 bpm. After being placed in Trendelenburg position, type and cross, quantitative βhCG, and repeat CBC were drawn.

**CPC-EM Capsule**

What do we already know about this clinical entity?

*Cornual pregnancy describes a rare, potentially disastrous implantation within the myometrium beside the fallopian tube, difficult to find on traditional ultrasound*.

What makes this presentation of disease reportable?

*This case illustrates a ruptured cornual pregnancy in the ED with severe hemorrhage, previously thought resolved by methotrexate via obstetrics*.

What is the major learning point?

*Given extended progression prior to diagnosis and disproportionate maternal mortality, cornual pregnancy should be a chief concern in suspected ectopic pregnancy*.

How might this improve emergency medicine practice?

*Cornual pregnancy is best diagnosed by three-dimensional ultrasound. Given high mortality from hemorrhage, all cases require emergent obstetrics consultation*.

**Image 2:**
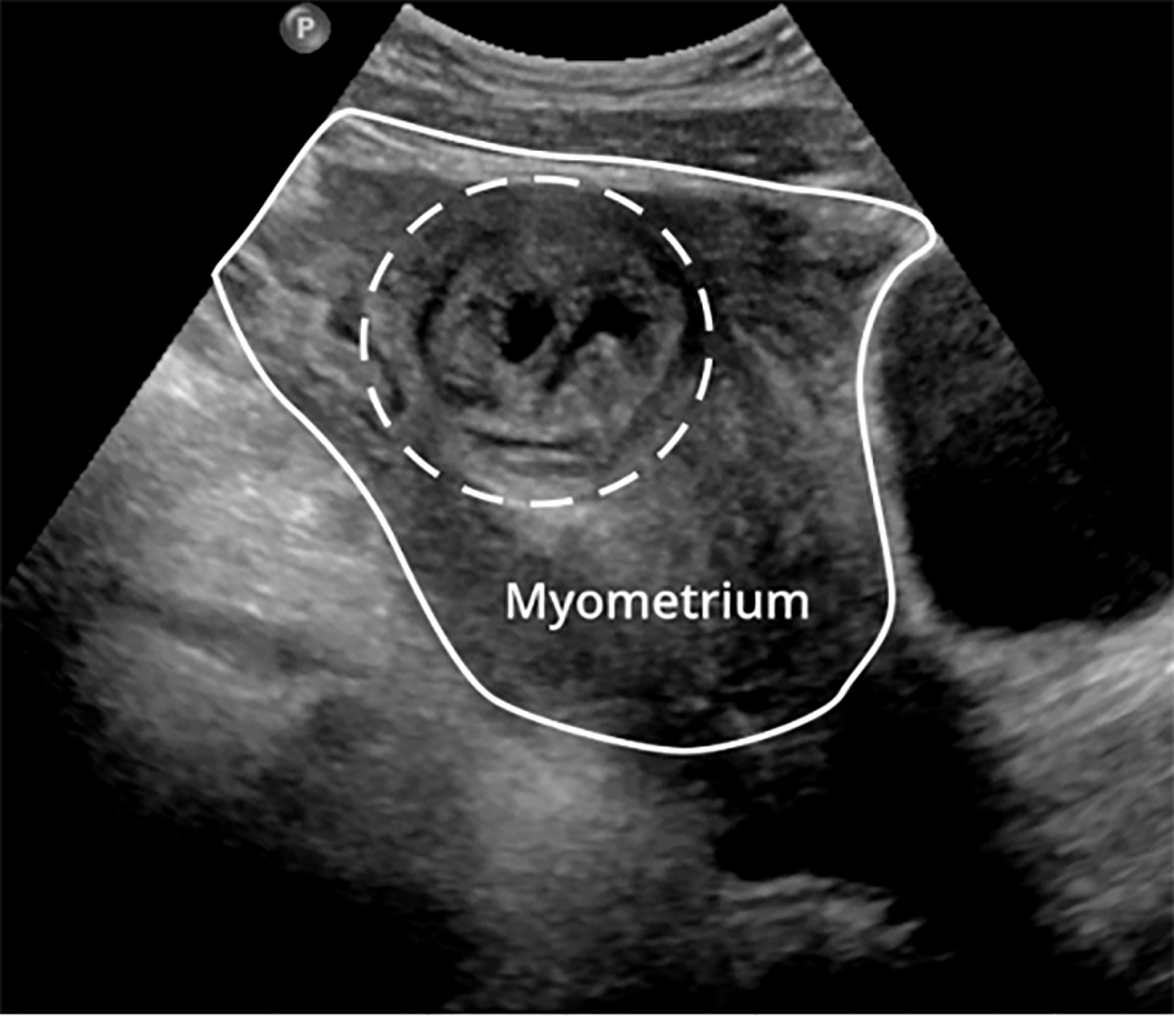
Transabdominal ultrasound demonstrating a mass in the left lateral uterus (dashed circle).

**Image 3:**
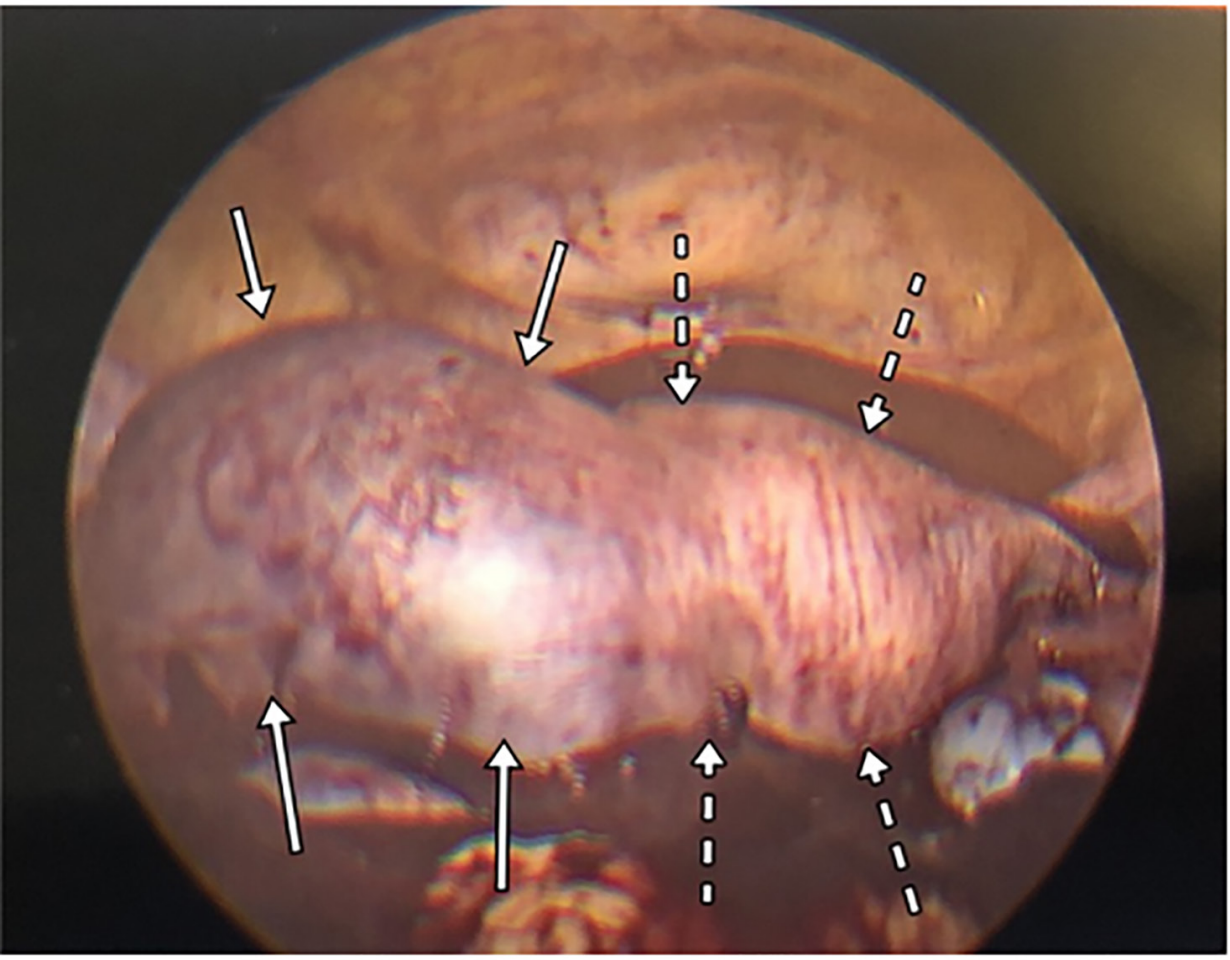
Laparoscopic image of visualized cornual pregnancy (solid arrow) and uterus (dashed arrow) surrounded by large hemorrhage.

Point-of-care US revealed free fluid in the abdomen as well as a thickened endometrium with an endometrial mass (Image 2). Obstetrics was emergently consulted for suspected ectopic rupture; upon arrival to bedside, repeat hemoglobin and hematocrit revealed a dramatic decline to 6.2 g/dL and 20.1%, suspected due to hemorrhage. Two units of packed red blood cells were administered en route to the operating room. The surgeons found approximately two liters of intraperitoneal blood and diagnosed a ruptured, left cornual ectopic pregnancy (Image 3). A cornual wedge resection and a unilateral salpingectomy were performed. The patient recovered uneventfully and was discharged on postoperative day three.

## DISCUSSION

In the past, a cornual pregnancy described implantation and development of a gestational sac in a bicornate or septate uterus. Today the term more broadly describes implantation in the myometrium of the horn (cornual region) of a normal uterus.^1^–^4^ Although cornual pregnancy is sometimes used interchangeably with interstitial pregnancy, the latter is distinguished by a gestational sac within the myometrium not specific to the cornual region.^1^–^5^ These constitute 2–4% of all ectopic pregnancies.^5^–^9^ Cornual pregnancy carries a 2.5% mortality rate but disproportionately accounts for 20% of maternal deaths from ectopic pregnancy.^4^,^7^,^10^

Compared to more common tubal pregnancies, the ability of the cornual uterine tissue to stretch allows pregnancies to progress undetected for weeks longer prior to rupture.^4^,^9^,^11^ Traditional ectopic pregnancy risk factors such as previous ectopic gravidity, pelvic inflammatory disease, fibroids, fallopian tube obstruction, and in vitro fertilization may be present but are largely absent in case series.^3^,^4^,^7^,^8^ Most ruptured cornual pregnancy patients complain of abdominal pain, but vaginal bleeding is less frequent than in other ectopic pregnancies, likely given the sequestered location.^4^,^8^,^9^ However, as pregnancy may progress as long as 12 weeks prior to rupture, women may present with hemorrhagic shock, confounding initial evaluation, differential considerations, and management.^3^,^9^,^12^

Cornual location within the interstitium can be confused with an uncomplicated intrauterine pregnancy on traditional two-dimensional US.^4^,^13^ A proposed, sonographic “interstitial line sign” extending from the upper region of the uterine horn to border the intramural portion of the fallopian tube has been described with high specificity but low sensitivity.^7^,^13^,^14^ Diagnostic criteria center on sonographic findings of an empty uterine cavity, a chorionic sac seen separately greater than one centimeter from the most lateral edge of the uterine cavity, and a thin myometrial layer surrounding the gestational sac.^8^,^13^,^15^ Like the interstitial line sign, these models demonstrate high specificity but low sensitivity of 40%.^14^ Radiologists and obstetricians advocate for three-dimensional US to improve diagnosis, as it can image the coronal plane of the uterus, although validation of increased accuracy is lacking.^3^,^4^,^12^,^13^

Suspicion of cornual pregnancy necessitates emergent obstetric consultation. Initial management focuses on stabilization and pregnancy confirmation. Unstable vital signs with free fluid on abdominal US should prompt consideration for blood product transfusion. In stable patients, early detection (βhCG < 3000 mIU/mL) of cornual pregnancy can be considered for outpatient methotrexate therapy, through oral, intramuscular, or sonographic-guided injection with obstetrics consultation.^4^,^8^,^13^ Most cited surgical cases are past this threshold, requiring either laparoscopic cornual resection or hysterectomy, although some are successfully treated with outpatient methotrexate.^4^–^9^,^13^

## CONCLUSION

Given the difficulties in imaging and extended gestational age prior to diagnosis of cornual pregnancy, and high mortality rates, it is important that emergency clinicians be familiar with diagnostic pitfalls and treatment plans for management. Proper resuscitation and supportive care in the ED setting are essential, and immediate obstetric surgical consult is critical to limit catastrophic hemorrhage.
